# Recent advances in the effects of microwave radiation on brains

**DOI:** 10.1186/s40779-017-0139-0

**Published:** 2017-09-21

**Authors:** Wei-Jia Zhi, Li-Feng Wang, Xiang-Jun Hu

**Affiliations:** Laboratory of Experimental Pathology, Beijing Institute of Radiation Medicine, Beijing, 100850 China

**Keywords:** Microwave, Central nervous system, Dysfunction of learning and memory abilities

## Abstract

This study concerns the effects of microwave on health because they pervade diverse fields of our lives. The brain has been recognized as one of the organs that is most vulnerable to microwave radiation. Therefore, in this article, we reviewed recent studies that have explored the effects of microwave radiation on the brain, especially the hippocampus, including analyses of epidemiology, morphology, electroencephalograms, learning and memory abilities and the mechanisms underlying brain dysfunction. However, the problem with these studies is that different parameters, such as the frequency, modulation, and power density of the radiation and the irradiation time, were used to evaluate microwave radiation between studies. As a result, the existing data exhibit poor reproducibility and comparability. To determine the specific dose-effect relationship between microwave radiation and its biological effects, more intensive studies must be performed.

## Background

Microwaves are electromagnetic waves with frequencies ranging from 300 MHz to 300 GHz. Microwaves are widely used in households, industry, communications, and medical and military buildings, and they provide substantial contributions to the development of human society. However, with its popularization, increasing attention has been paid to its influence on humans. Electromagnetic radiation can be absorbed by organisms, in which it causes a series of physiological and functional changes. Many intricate electrical activities occur in the central nervous system, including learning and memory, which are therefore vulnerable to electromagnetic radiation. Moreover, the popularization of mobile phones has made them the main source of brain exposure to radiation. Therefore, the central nervous system is considered one of the most sensitive organs that is targeted by microwave radiation [1, 2]. A large number of studies have shown that microwave radiation can cause a series of adverse reactions in the central nervous system, including sleep disorders in addition to learning and memory impairments.

Microwaves are widely used in broadcasting, communications and many industrial fields. In broadcasting, the sources of microwaves are mainly FM radio and TV broadcasting antennas, which produce frequencies ranging from 80 to 800 MHz. In communications, the microwaves come from mobile phones and their base stations and microwave links, in addition to cordless phones, terrestrial trunked radios, blue tooth devices, wireless local area networks and many other applications. The frequencies of these devices are listed in Table 1. In industrial fields, exposure is usually occupational, and its sources include the surgical and physiotherapeutic use of diathermy, dielectric heating (i.e., heating and vulcanization applications), microwave ovens, magnetic resonance imaging (MRI) medical diagnostic equipment, radar, military and research microwave systems, electricity-supplying networks, and electricity-distributing and transmitting equipment [3].

**Table 1:** Source and frequency range of microwave

Application	Frequency range (MHz)
FM radio and TV broadcasting antennas	80–800
Mobile phones	453.5–1980.0
Mobile phone base station	463.5–2170.0
Microwave links	1000
Cordless phones	1880–1900
Terrestrial trunked radio	380–470
Bluetooth devices	2450
Baby monitors	40, 446, 864, 1900 and 2450
Wireless local area networks	2400 and 5000
Smart meters	900–1900 or 2400
Surgical and physiotherapeutic	2450
Diathermy	2450 and 434
Microwave ovens	915–2450
Radar	30–300,000

Based on this background, in this review, we first summarized the effects of microwave radiation on the central nervous system, including the epidemiology, morphology, electroencephalograms, learning and memory abilities and mechanisms of underlying brain dysfunction from the perspective of synaptic structures and functions, oxidative stress and apoptosis, protein synthesis, genes and individual susceptibility and energy metabolism.

## Epidemiology

In 2011, the International Agency for Research on Cancer (IARC) announced that microwave radiation has potentially carcinogenic effects (2B). However, it concurrently also declared that the carcinogenic potential of mobile communications equipment was limited to glioma [4].

### Exposure to mobile phones

Of the numerous studies performed to explore the effects of mobile communication devices on humans, only a few have shown that cell phones and brain tumors are statistically correlated. For example, people who have used mobile phones for more than 10 years have a clearly higher risk of brain tumors. Those who are accustomed to using their mobile phone ipsilaterally presented a probability that was twice that of people who don’t [5–7]. However, most studies have not supported the conclusion that cell phones cause brain tumors [8–12]. One study reported by the Interphone study group [13] showed that there was no increase in the risk of glioma or meningioma in users of mobile phones. It has been suggested that there is an increased risk of glioma at the highest exposure levels, but biases and errors prevent causal interpretations of these data. Additionally, Larjabaara et al. [14] found that gliomas are not preferentially located in the parts of the brain with the highest exposure. Finally, Hardell et al. [15] assessed the use of mobile and cordless phones in 347 cases of melanoma in the head and neck region and 1184 controls and found no increased risk.

### Occupational exposure

#### Industrial exposure

In long-term epidemiological investigations of large population with occupational exposure, the results have not been consistent. Dasdag et al. [16] investigated workers who worked at a television transmitter station with a frequency ranging between 202 and 209 MHz, 694–701 MHz, 750–757 MHz, or 774–781 MHz and at a medium-wave broadcasting station. Their answers to questionnaires showed that the workers suffered from symptoms including headaches, fatigue, stress and sleeplessness. Most of the workers recovered when they left the source of microwave radiation. In addition, another study showed that significant psychiatric symptoms were observed in people who worked in these areas. In particular, somatization, obsessive compulsivity, paranoid ideation and psychoticism were reported [17].

#### Military exposure

Standard devices used by military personnel that may pose electromagnetic hazards include radars and missile systems. In a report by the Poland Department of Microwave Safety, occupational exposure to electromagnetic fields was analyzed in the work environment of personnel of 204 devices divided into 5 groups (surface-to-air missile system radars, aircraft and helicopters, communication devices, surveillance and height finder radars, airport radars and radio navigation systems). In 57% of military devices, Polish soldiers work in occupational protection zones. In 35% of cases, soldiers work in intermediate and hazardous zones and in 22%—only in the intermediate zone. In 43% of devices, military personnel are not exposed to an electromagnetic field.

The visual reaction time and short-term memory of healthy male and female workers at a radar site with a frequency range of 2–18 GHz was recorded with a simple blind computer-assisted-visual reaction time test or modified Wechsler Memory Scale test. The results indicated that radar microwave radiation leads to a decreased reaction time and lower short-term memory performance [18]. Among radar workers exposed to 14–18 GHz microwaves, the somatic symposium anxiety and insomnia, social dysfunction and severe depression were caused [19]. Singh et al. [20] divided the radar workers into three sets: control group (*n* = 68), exposure group I (*n* = 40, exposed to 8–12 GHz) and exposure group II (*n* = 58, working with radar at 12.5–18.0 GHz). The three groups were further divided into two groups according to their years of service (up to 10 years and >10 years) to investigate the effect of years of exposure to radar. Melatonin and serotonin levels were estimated, which play important roles in the nervous system. The results demonstrated the ability of electric magnetic field (EMF) to influence plasma melatonin and serotonin concentrations in radar workers. The results were significant for the range from 12.5–18.0 GHz with a service period greater than 10 years. Additionally, people exposed to military microwave sources were more vulnerable to brain tumors. Richter et al. [21, 22] found a higher incidence of brain cancer in radar technicians and a shortened incubation period (i.e., less than 10 years). Szmigielski [23] collected retrospective data for Polish soldiers over 15 years and showed that the prevalence of brain cancer was higher in each age group.

### Effects of microwave radiation on children

Because a child’s nervous system is growing and their head is more vulnerable to radiation energy, studies that have specifically addressed whether the nervous systems of children are more susceptible to electromagnetic radiation have been performed. However, there is little scientific evidence to demonstrate that children are more sensitive to electromagnetic radiation than adults [24, 25].

### Positive effects

With the increasing number of applications that use microwave technology, its negative effects on the human body have attracted attention. However, its beneficial effects should not be ignored [26]. For example, it can shorten reaction times so that people can better cope with danger. Mortazavi et al. [27] found that college students’ visual reaction times were significantly shorter after 10 min of phone-induced microwave radiation. This conclusion is consistent with the results of a previously reported study that showed that short-term exposure to microwave radiation can reduce reaction times and improve cognitive functions, attention and short-term memory capacity [28–34]. Moreover, the risk of developing Alzheimer’s disease is 30–40% lower in people who use a mobile phone for more than 10 years than in other individuals [35]. The above results indicate a positive biological effect of microwave radiation and present a challenge with regard to how people can benefit from microwave radiation.

Above all, because of biases and variations in investigation methods, no conclusive evidence has been presented that microwaves cause cancer. We should ensure that we avoid excessive exposure to microwave radiation in daily life activities and use mobile phones appropriately despite information about its positive effects. In the population with occupational exposure, proper protective measures should be taken to avoid unnecessary harm. These conclusions are presented in Table 2.

**Table 2:** Epidemiological studies of microwaves

Item	Reference	Sample/Model	Exposure condition	Results
Occupational exposure	Dasdag et al. 1992	Workers in TV transmitting station and medium wave broadcasting station	Frequency ranging between 202 and 209, 694–701, 750–757, 774–781 and 1062 MHz	Suffered from many illnesses
	Dasdag et al. 1994	Workers in TV transmitting station and medium wave broadcasting station	Frequency ranging between 202 and 209, 694–701, 750–757, 774–781 and 1062 MHz	Showed significant psychiatric signs
	Dasdag et al. 1999	Radio-link technicians and other workers, working years of subjects were 10 to 22 years	167 MHz, 420 MHz, 2 GHz and 6 GHZ with output powers of 200.0, 10.0, 1.5 and 4.0 watt, respectively	Fatigue, headache, irritability, loss of appetite, sleepiness and memory difficulties were observed, but there were no pathological findings
	Richter et al. 2002	5 young patients had brain tumors, 10 years of initial occupational exposures to radar	GHz range; 10-300 W	Incubation period shortened
	Szmigielski et al. 1996	Military career personnel in Poland during a 15-year period (1971–1985)	Pulse-modulated microwaves at 150–3500 MHz) 2–6 W/m^2^	Prevalence of brain cancer in each age group was higher
Mobile phone exposure	Interphone study group. 2010	Regular mobile phone user	Mobile phones	No increase in risk of glioma or meningioma was observed. An increased isk of glioma at the highest exposure levels was observed, but biases and error prevent a causal interpretation
	Larjabaara et al. 2011	Regular mobile phone user	Mobile phones	Gliomas are not preferentially located in parts of the brain with the highest exposure
	Hardell et al. 2011	2708 glioma and 2409 meningioma cases	Mobile phones	No increased risk
	Carlberg et al. 2013	Brain tumor cases of both genders aged 18–75 years and diagnosed during 2007–2009	Mobile phones	No conclusive evidence of an association between use of mobile phones and cordless phones and meningioma
	Mortazavi et al. *2*014	College students	900 MHz GSM mobile phone; 10 min	Visual reaction time decreased
	Koivisto et al. 2000	Healthy humans	902 MHz electromagnetic field emitted by cellular telephones	Reaction time reduction
	Preece et al. 2009	Humans	915 MHz	Improvement of cognitive function
	Koivisto et al. 2000	Healthy subjects	902 MHz electromagnetic field emitted by GSM phones	Improvement of working memory
	Edelstyn et al. 2002	Healthy subjects	Electromagnetic field emitted by a 900 MHz mobile phone for 30 min	Improvement of attentional capacity and processing speed
	Smythe et al. 2003	Male and female subjects	Mobile phones	Spatial learning ability were improved in males but not in females
Effects on children	Otto et al. 2007	Children	Mobile phones	Microwaves encountered in common life are most likely not a priority issue in children’s environmental health
	Aydin et al. 2011	352 families of brain tumor patients in 7–19 years old adolescents and 646 matched controls	Mobile phones	Non-significantly increased OR value
	Lee et al. 2001	Adolescents	Electromagnetic field emitted by mobile phones	Mild facilitating effect on attention functions

## The influence of microwave radiation on the central nervous system

### Negative effects

A popular focus among researchers is the damage that microwave radiation causes in the central nervous system, in which it can impair learning and memory.

The Morris water maze has been widely used in neurobehavioral tests [36]. This classical method is often used to test learning and memory abilities after exposure to microwave radiation. Sareesh et al. [37] exposed male Wistar rats (10–12 weeks old) to a Global System for Mobile Communication (GSM) (900/1800 MHz) mobile phone that was in vibrating mode (i.e., no ring tone). In the treated rats, 50 calls were missed each day for 4 weeks, and the spatial memory abilities of the rats were tested after the experimental period. They found that escape times were substantially decreased when the animals were trained while exposed to the phone. In the probe test, the exposed group could not locate the platform and exhibited a significantly higher mean latency (i.e., 3-fold higher) to reach the target quadrant, and they spent only half the time that the controls spent in the target quadrant. These results indicated that mobile phone exposure affected the acquisition of learned responses in Wistar rats. Wang et al. [38] exposed Wistar rats to a 2.856 GHz pulsed microwave field for 6 min. The fields had an average power density of 0, 5, 10 or 50 mW/cm^2^. The results showed that at 6 h, 1 d and 3 d after exposure, the groups in which the average power density was 10 mW/cm^2^ or 50 mW/cm^2^ displayed significant deficits in spatial learning and memory. Additionally, the number of crossings was significantly lower at 3 d after microwave radiation.

### Neutral effects

Researchers have also used the 12-arm maze test on rats to test spatial working ability after exposure to 2.45 GHz pulse microwave radiation. Lai et al. [39] exposed rats to microwaves (500 pps, pulse width = 2 μs, and average whole body-specific absorption rate (SAR) = 0.6 W/kg) for an exposure duration of 45 min, and a significant decline was observed in the rats’ performance, indicating that microwave radiation influenced their working memory. However, these experimental results remain to be supported in repeated experiments [40–42]. Cassel et al. [41] exposed rats to 2.45 GHz microwaves (2 μ pulse width, 500 pps, and SAR 0.6 W/kg) for 45 min and found that microwave-induced behavioral alterations measured by Lai had more to do with factors related to performance bias than to spatial working memory. Cosquer et al. [40] also repeated the experiment only and found that radial-arm maze performance in rats remained unchanged. Cobb et al. [42] found similar results. Thus, Cosquer et al. [40] concluded that despite the differences in the conditions used in the experiments, space limitations and whether the wall of the maze was clear did not substantially influence outcomes.

Positive results were obtained in Morris water maze tests that demonstrated that microwaves influenced learning and memory in rats. However, these effects were not observed in radial arm maze tests. One reason for this difference may be that the water maze experiment is driven by aversion, whereas the arm maze experiment is driven by appetite. The former is now recognized worldwide as a method for evaluating learning and memory, while the latter is viewed as more susceptible to other factors. The conclusions are shown in Table 3.

**Table 3:** The influence of microwave radiation on learning and memory

Item	Reference	Method	Sample/Model	Exposure condition	Results
Negative effects	Sareesh et al. 2009	MWM	Male Wistar rats (10–12 weeks old)	50 missed calls/day for 4 weeks from a GSM (900/1800 MHz) mobile phone in vibratory mode (no ring tone)	Mobile phone exposure affected the acquisition of learned responses in Wistar rats
	Wang et al. 2013	MWM	Male Wistar rats	2.856 GHz pulsed microwave field for 6 min (unexposed, 5, 10 and 50 mW/cm^2^)	10 and 50 mW/cm^2^ displayed significant deficits inspatial learning and memory
	*Lai* et al. 1994	12 radial-arm maze	Rats	Exposure to pulsed 2450 MHz microwaves for 45 min	Deficit in spatial “working memory” function
Neutral effects	Cassel et al. 2004	12 radial-arm maze	Rats	2.45 GHz microwaves (500 pps, pulse width = 2 μs, average whole body SAR = 0.6 W/kg for 45 min)	Microwave-induced behavioral alterations measured by Lai had more to do with factors related to performance bias than to spatial working memory
	Cosquer et al. 2005	12 radial-arm maze	Rats	Whole-body exposure to 2.45 GHz electromagnetic fields	Radial-arm maze performance in rats did not changed
	Cobb et al. 2004	12 radial-arm maze	Rats	45 min exposure to 2450 MHz fields at whole body SARs of 0.6 W/kg (500pps, pulse width = 2 μs); pre-exposure injection of one of three psychoactive compounds or saline	Exposure to microwave radiation did not cause decrements in the ability of rats to learn the spatial memory task

*GSM* Global system for mobile communication, *MWM* Millimeter wave mixer

## The influence of microwave radiation on the morphology of the brain

The central nervous system, especially the hippocampus, is highly sensitive to microwave radiation [43, 44]. Previous studies have shown that in unexposed control rats, hippocampal neurons are aligned in neat rows in which the edges are clear, nuclei are clear, nucleoli can be observed, and pyramidal cells do not exhibit obvious necrosis. However, in rats treated with long-term exposure to radiation, neurons exhibit edema and are arranged irregularly. Nuclear pyknosis and capillary congestion are also observed.

Regarding the ultra-structure of the hippocampus, symptoms including neuronal atrophy, mitochondrial swelling, crest reduction and a disordered arrangement were observed, the rough endoplasmic reticulum exhibited cystic expansion, the number of synaptic vesicles decreased, and the synaptic cleft was widened (2.45 GHz pulsed microwave field at an average power density of 1 mW/cm^2^ for 3 h/d for up to 30 days [45] and an average power density of 2.5, 5, or 10 mW/cm^2^ for 6 min/d for up to 1 month resulted in an average calculated SAR of 1.05, 2.1, and 4.2 W/kg, respectively [46]). The hippocampus plays roles in learning and memory, and the results of these studies suggest that the deficits in learning and memory functions observed after exposure to microwave irradiation might be due to abnormalities induced in hippocampal structures.

## The influence of microwave radiation on electroencephalograph (EEG) data

Brain electrical activity originates from the membrane potential of the neuron itself and the fluctuation in membrane potential. The transduction of a nerve impulse and the postsynaptic potential produced by it result in synaptic transmission. EEG data reflect the functional state of the brain by enlarging the autologous weak bioelectricity recorded by the EEG-recording instrument [47]. An abnormal EEG is closely associated with damaged cognitive ability. EEG is often used as a tool to diagnose Alzheimer’s disease [48]. Most studies have suggested that microwave radiation can cause EEG abnormalities in experimental animals and participants, but some negative results have also been reported in studies using low-power microwaves.

Vorobyov et al. [1] used 10 freely moving rats in which carbon electrodes were implanted in the cortex and dorsomedial hypothalamus. Of these, five rats were repeatedly exposed to extremely low-frequency microwaves (915 MHz; pulse width, 20 ms; average power density, 0.3 mW/cm^2^; repetition frequency, 4.0 Hz; intermittently for 1 min, ‘On’ for 1 min, and ‘Off’ for 10 min; SAR, 0.7 mW/g) and 5 were in the sham group. The authors detected the EEG within the frequency bands of 0.5–30.0 Hz for 5 consecutive days. The results showed that in normal EEGs, the θ (3.2–6.0 Hz) and β_2_ (17.8–30.5 Hz) waves were mainly concentrated in the cortex, while the α (6.0–17.8 Hz) waves were mainly concentrated in the hypothalamus. After exposure, the levels of β_2_ waves in the hypothalamus increased more than those in the cortex, leading to a significant reduction in the deviation of the two EGGs. The results indicated that repeated low-level exposure to extremely low frequency microwaves affects brain functioning and provide an additional approach to analyzing the underlying mechanisms.

In a study investigating the influence of low-frequency microwave (450 MHz) radiation on EEG, Hinrikus et al. [49] found that microwave radiation enhanced brain wave energy, decreased brain wave frequency, and increased the amplitude and power of delta frequency bands, indicating a decrease in learning ability [50–53]. More data are available regarding opinions on potential health effects of exposure to electromagnetic fields [3].

Many recent studies have reported that microwaves exposure affects EEG results [54–57]. Suhhova et al. [58] exposed volunteers to microwaves at a frequency of 450 MHz for 10 repeated intervals of 1 min of irradiation and 1 min off. The SAR of the two groups were 0.303 W/kg and 0.003 W/kg. A resting eyes-closed electroencephalogram was used to continuously record the results, which showed that there was an increase in the power of the α, β_1_ and β_2_ frequency bands in the 0.303 W/kg group and in the β_2_ frequency bands in the 0.003 W/kg group. Statistically significant changes were detected in the EEG-α bands of six individuals and in the β_1_ and β_2_ bands of four subjects in the higher SAR group. In the lower SAR group, the α, β_1_ and β_2_ bands were affected in the three subjects. This study also revealed the dose-dependent relationship of the modulated microwave effect: decreasing the SAR 100-fold reduced the associated changes in the EEG by three- to six-fold and decreased the number of affected subjects but did not completely eliminate the effects.

## The influence of microwave radiation on postnatal development

Maternal exposure to Wi-Fi radio frequencies led to various adverse neurological effects in the offspring. Othman et al. [59] exposed Wistar albino pregnant rats to a 2.45 GHz Wi-Fi signal for 2 h/d throughout the gestation period and found that the neurodevelopment, cerebral stress equilibrium and cholinesterase activity of the offspring were affected. To investigate the potential combined influence of maternal restraint stress and 2.45 GHz Wi-Fi signal exposure on postnatal development and behavior in the offspring of exposed rats, control, Wi-Fi-exposed, restrained and both Wi-Fi-exposed and restrained groups were established. Each Wi-Fi exposure and restraint occurred for 2 h/d during gestation until parturition. The results showed that gestational Wi-Fi exposure and restraint adversely affected offspring neurodevelopment and behavior in adulthood. The progeny brain oxidative balance and serum biochemistry, such as phosphorus, magnesium, glucose, triglycerides and calcium levels, were disrupted [60].

Zhang et al. [61] found that after pregnant rats were irradiated with 9.417 GHz microwaves, the behavior of their offspring differed, and the outcome was sex-dependent. An increase in anxiety-related behaviors and a decrease in depression-related behavior were observed in both female and male offspring. However, impaired learning and memory were only observed in males. Zhang proposed that the sex-dependent relationship between microwaves and the behavior of offspring may be related to sex hormones, and female rats may be equivalently protected by reducing oxidative stress levels.

## Mechanisms underlying learning and memory are damaged by microwave irradiation

### Synaptic structures and functions

Synapses are special structures that are involved in the transmission of electrochemical signals between neurons in the central nervous system. Synaptic plasticity is a special function of synapses, which play an important role in learning and memory processes [46], including structural and functional plasticity. After exposure to microwave radiation, during synaptic structural plasticity, presynaptic vesicles accumulate or empty, mitochondria are damaged, postsynaptic membranes are perforated, postsynaptic lengths and postsynaptic density distributions are abnormal, mossy fiber growth is inhibited during learning and memory functions, dendritic filopodial densities and activities are decreased, and there is a significant reduction of the dendritic spine density and dendritic fragment length [62]. Functional plasticity is affected in other ways, including the abnormal release and uptake of brain amino acids such as choline and monoamine neurotransmitters, a decrease in excitatory postsynaptic potential amplitudes and spikes in long-term potentiating (LTP) in the medial perforated pathway (MPP) in the dentate gyrus (DG) [27].

#### In vitro studies

Ning et al. [63] exposed rat hippocampal neurons to microwaves (SAR values of 0.8 W/kg and 2.4 W/kg at an average power density of 1800 MHz/d) and observed the formation of dendritic filopodial and dendritic branches and the maturation of dendritic spines in neurons from day 6 to 14. Additionally, in the 2.4 W/kg group, neuronal filopodial density and activity were lower on day 8, and there was a reduction of the dendritic spine maturity on day 14. In the group treated with 0.8 W/kg, there was no significant change. Thus, in the early developmental stage, chronic exposure to 2.4 W/kg GSM microwaves may influence dendritic development and the formation of excitatory synapses in cultures of hippocampal neurons. Xu et al. [64] exposed cultured rat hippocampal neurons to GSM 1800 MHz microwaves (SAR, 2.4 W/kg) and observed a selective decrease in the amplitude of α-amino-3-hydroxy-5-methyl-4-soxazole propionic acid (AMPA) miniature excitatory postsynaptic currents (mEPSCs). However, there was no change in the frequency of AMPA mEPSCs or the amplitudes of N-methyl-d-aspartate (NMDA) mEPSCs. Furthermore, the expression of postsynaptic density 95 (PSD-95) in cultured neurons was decreased. Thus, these results suggest that the 2.4 W/kg GSM 1800 MHz microwaves may reduce excitatory synaptic activity and the number of excitatory synapses in cultured rat hippocampal neurons.

#### In vivo studies

Wang et al. [65] irradiated Wistar rats with 10, 30 and 50 mW/cm^2^ microwaves, and the results showed that in the cerebral cortex, only glycine (Gly) and asparagine (Asp) levels were increased. Microwaves of 50 mW/cm^2^ increased the levels of the major excitatory amino acids Asp and glutamic acid (Glu) and the inhibitory amino acids gamma-aminobutyric acid (GABA) and Gly, while 6 h later in the 30 mW/cm^2^ group, the level of Gly was reduced in the cerebral cortex. However, Li et al. [50] exposed Wistar rats to 2.856 GHz microwaves at an average power density of 5, 10, 20 or 30 mW/cm^2^ for 6 min three times per week for up to 6 weeks and found that on day 14 after irradiation, the levels of Asp and Glu were lower in the hippocampus in the group treated with 5 mW/cm^2^ but higher in the group treated with 30 mW/cm^2^, The levels of GABA were elevated. After 28 days, the levels of Glu and Tau in the hippocampus and cerebrospinal fluid were lower, indicating that the cognitive damage induced by microwave radiation is associated with a decrease in Glu [66, 67].

Synaptic vesicles form in different parts of neurons and contain high concentrations of substances that are transferred from the neuron. When nerve endings are excited, the vesicles release their contents into the synaptic cleft, resulting in synaptic transmission. The normal function of synaptic vesicles depends on the normal expression of related proteins. Wang et al. [68] radiated Wistar rats with microwaves (30 mW/cm^2^; SAR value, 14.1 W/kg), and then detected the expression of synaptic vesicle-associated proteins and found that synaptophys in I and VAMP-2, which are synaptic fusion proteins, and synaptic vesicle proteins were abnormally expressed to different degrees. The authors therefore proposed that synaptic conduction disorders are associated with damage to cognitive functions. Qiao et al. [62] irradiated Wistar rats, hippocampal synaptosomes and differentiated PC12 cells using microwaves (average power density, 30 mW/cm^2^) for 5 min, and the results showed that the post-exposure spatial memory of the rats was significantly decreased, the post-exposure levels of phosphorylated synapsin I (p-synapsin I) and GABA were decreased in the rat and cell experiments, and the post-exposure levels of vesicular GABA transporter and p-synapsin I were increased in small clear synaptic vesicles (which were abnormally assembled in presynaptic terminals) in the rat experiments. Exposure to microwaves and silencing p-synapsin I reduced the release of GABA, and maximum reduction was achieved when both were combined, indicating a synergistic effect. Xu et al. found that long-term treatment with a low dose of microwave radiation reduced the activity and the number of excitatory synapses.

### NMDA receptors

Among the variety of neurotransmitters, glutamate is the most abundant endogenous amino acid in the mammalian central nervous system. It influences both learning and memory in rats [67]. In the CNS, glutamic acid binds and plays physiological roles with the following two receptors: ionotropic glutamate and metabolic glutamate. The ionotropic receptors consist of NMDA receptors and non-NMDA receptors.

N-methyl-D-aspartic acid receptor (NMDAR) is a tetramer composed of two NR1 and two NR2 subunits or two NR3 subunits that perform the functions of NMDAR [69, 70], and NMDAR plays key roles in synapse development, synaptic plasticity and neurological diseases. LTP induction involves a signal transduction cascade that includes the release of glutamate from synaptic vesicles, activation of NMDAR at postsynaptic membranes, entry of Ca^2+^, and activation of Ca^2+^/calmodulin-dependent protein kinases (CaM kinases) II, IV and mitogen-activated protein kinase (MAPK) [71]. Xiong et al. [46] found that by excessively activating the NMDA receptor signaling pathway, microwaves undermine hippocampal synaptic plasticity, explaining the damage observed in learning and memory abilities in radiated rats. Wang et al. [72] found that 2.856 GHz, 50 mW/cm^2^ pulsed microwave radiation caused persistent spatial memory impairments, disordered neurotransmitters, and varying degrees of damage in the hippocampus and synapses. The levels of NMDA receptor subunits were increased 1 month after irradiation. NR2B plays a key role in LTP and was decreased from the 3rd to the 18th month post-treatment, and long-term exposure to high doses of radiation may therefore damage cognitive functions. This effect is similar to the decrease observed in NR2B in rats. It has also been reported that acute exposure to continuous waves of 900 MHz EMF or 900 MHz waves that were modulated to an amplitude of 50 Hz increased reactive oxygen species (ROS) levels and DNA fragmentation as a result of the hyperstimulation of glutamate receptors [73].

### Oxidative stress and apoptosis

Oxidative stress refers to an imbalance between oxidants and antioxidants in vivo and is characterized by a biochemical state that tends toward oxidization, including the formation of oxygen free radicals (i.e., ROS) and nitrogen radicals reactive nitrogen species (RNS), which play major roles in oxidation. Numerous mechanisms can activate oxidative stress, including electromagnetic radiation, and thereby cause molecular damage. This damage plays a key role in the structural and functional changes that are accelerated by neuronal degeneration. It has been reported that microwave radiation can induce lipid peroxidation of cell membranes and produce apoptotic signals [74, 75]. Microwave radiation can induce oxidative and nitrosative stress, which lead to hippocampal neuronal and non-neuronal apoptosis via the oxidative damage of cellular constituents (i.e., nucleic acids, proteins and lipids) and subsequent over expression of p53, which up-regulates Bax and down-regulates pro-caspase-3 and full-length/uncleaved poly-ADP-ribose polymerase (PARP) 1, eventually inducing neuronal degeneration via apoptosis [76]. Chronic microwave exposures were executed with 2.45 GHz of either modulated (power density, 0.029 mW/cm^2^; specific absorption rate, 0.019 W/kg with a sinusoidal modulation of 400 Hz) or non-modulated continuous sinusoidal wave (power density, 0.033 mW/cm^2^; specific absorption rate, 0.023 W/kg) for 2 h daily for 1 month. The results suggested that chronic non-modulated, but not modulated, microwave radiation may cause anxiety-like and depression-like behaviors and calcium- and NO-related biochemical changes in the brain [77].

#### In vitro studies

Shahin found that regardless of whether exposure was long-term or short-term, 2.45 GHz microwaves increased oxidative/nitrosative stress, which potentially led to apoptosis in hippocampal subfield neurons and non-neuronal cells as a result of p53-dependent/−independent activation. Mack et al. [78] exposed differentiated astroglial cells that were cultured for 14 days in vitro to either continuous 900 MHz waves or 900 MHz waves modulated in amplitude at 50 Hz using a sinusoidal wave form and 100% modulation index for 5, 10, or 20 min. The strength of the electric field (rms value) at the sample position was 10 V/m. A significant increase in ROS levels and DNA fragmentation were observed only after the astrocytes were exposed to modulated EMF for 20 min, perhaps as a result of hyperstimulation of glutamate receptors. To investigate the effects of microwaves radiation on apoptotic activity, cell viability, and cell cycle progression, which can provide information about microwaves radiation effects on neural cells over the period from embryonic stages to infants. Human SH-SY5YNB cells were exposed to 2.1 GHz W-CDMA modulated microwave radiation for 24 h at a specific absorption rate of 0.491 W/kg. The results showed that 2.1 GHz W-CDMA-modulated microwave radiation did not cause apoptotic cell death but altered cell cycle progression [79].

#### In vivo studies

Joubert et al. [80] found that irradiation with 900 MHz, 2 W/kg continuous microwaves for 24 h induced an increase in rat neuronal apoptosis. Motawi et al. [81] reported a study exploring the influence of mobile phone microwave radiation on oxidative stress and apoptosis in rat brains. The experimental rats were divided into six groups of 3 adult rats and 3 young rats in each group (with control, GSM alone and call-receiving subgroups in each group). After irradiation was applied for 2 h/d for 60 d, the authors observed the following: microwave radiation produced by mobile phones damaged the brains of adult and young rats, the damage caused by mobile phones in the calling state was significantly more severe than that observed in the standby group, and the neurons of young rats were more seriously injured than those of adult rats. The direct cause of the observed neuronal damage may have been apoptosis induced by the microwave radiation, and an indirect cause may have been an increase in permeability of the blood-brain barrier (BBB), which would allow the traversal of toxic substances that could cause damage. Dasdag et al. [82] exposed animals to 900 MHz microwave radiation for 2 h/d for 10 months and removed the brain tissues. The final apoptosis score in the exposed group was significantly reduced, and the total antioxidant capacity and catalysis observed in the experimental group was increased. Therefore, the authors concluded that exposure to a radiofrequency of 900 MHz might trigger the neoplastic process because it produces a relative increase in the number of potentially long-lived cells.

### Protein synthesis

It is widely accepted that protein synthesis occurs in neuronal dendrites and may be the cellular basis of learning and memory, during which local protein synthesis and synaptic plasticity are closely linked to the efficiency of communication between neurons. The effects of microwave radiation on protein synthesis in brain remain undetermined.

Fragopoulou et al. [83] found that long-term exposure to microwave radiation (typical mobile phone, at an SAR level range of 0.17–0.37 W/kg for 3 h daily for 8 months, or wireless digital enhanced cordless telecommunications/telephone (DECT) base at an SAR level range of 0.012–0.028 W/kg for 8 h/d for 8 months) induced the synthesis of 143 proteins, including some neuronal function-associated proteins such as glial fibrillary acidic protein (GFAP), glial maturation factor (GMF), apolipo protein E, heat-shock protein, cytoskeletal proteins and some proteins that are associated with metabolism in the brain.

Verma et al. [84] and Sharma et al. [85] found that protein levels were reduced in rat brains following microwave radiation, which may have been caused by the excessive consumption or a reduction of the synthesis of proteins, and reduced protein synthesis can be caused by the following processes: (1) excessive activation of RNA enzymes; (2) mRNA consumption or the formation and maturation of RNA enzymes. Microwave radiation can cause tissue damage by inducing localized microstructural damage to proteins [86, 87]. It has been reported that microwave radiation can decrease both the number and the density of dendritic spines [63]. Dendritic spines, which are small protrusions that extend from dendritic shafts, are also cellular compartments containing signaling molecules that are important for synaptic transmission and plasticity [88–91]. These spines have been reported to share a close relationship with learning and memory abilities, and when protein synthesis in dendritic spines is blocked, new spine growth and the development of long spines are both decreased [91].

Dasdag et al. [92] found that 900 MHz microwave radiation emitted by mobile phones increased protein carbonyl levels in the brains of rats, suggesting that 900 MHz microwave radiation can alter some biomolecules such as proteins.

In summary, there is evidence that microwave radiation can lead to alterations in protein synthesis or protein modifications; however, the results are controversial. This phenomenon may be explained by the varied radiation dosage adopted in these studies.

### Genes and individual susceptibility

miRNAs are non-coding sequences with a length of approximately 22 nucleotides that have roles in cell development and differentiation and are also linked to signal transduction and tumorigenesis. Some studies have proposed that miRNAs play important roles in nerve regeneration, neurodegenerative diseases, and the pathogenesis of neuroblastoma and schizophrenia [93–95]. More than 50% of miRNAs are found in cancer-associated regions of the genome or in fragile sites, suggesting that miRNAs also play important roles in the pathogenesis of neoplasias [96]. Dasdag et al. [97] found that long-term exposure to 900 MHz radiation decreased the level of rno-miR107and that the whole body (rms) SAR value was 0.0369 W/kg, bridging the gap in the interaction between radio frequency radiation (FR) and miRNAs. Studies have also suggested that long-term exposure to 2.4 GHz microwave radiation may lead to adverse effects, as observed in neurodegenerative diseases that originate from the altered expression of some miRNAs. The authors found that 2.4 GHz microwave radiation reduced the expression of some miRNAs such as miR-106b-5p and miR-107 [98]. Zhao et al. [99] used a microarray and quantitative real-time PCR to analyze the miRNA expression profile in the hippocampus on days 7 and 14 after irradiation with a microwave at 30 mW/cm^2^. The authors predicted the differential expression of genes associated with transcription, translation and receptor functions (in addition to brain-related and signaling pathway-related) using the iRDB, miRbase and miRanda databases. They summarized the characteristics and functions of hippocampus-related miRNAs following irradiation with microwaves, and these data laid a foundation that clarified the molecular mechanisms underlying microwave-induced injury to hippocampal learning and memory and suggested potential therapeutic targets. To investigate the effects of 2.4 GHz Wi-Fi radiation on multisensory integration in rats, a cross-modal visual-tactile object recognition (CMOR) task was performed by four variations of the spontaneous object recognition (SOR) test including the standard SOR, tactile SOR, visual SOR, and CMOR tests. The results of this study showed that chronic exposure to Wi-Fi electromagnetic waves might impair both unimodal and cross-modal encoding of information. The increase in M1 receptor gene expression along with the impairment of novel preferences in Wi-Fi-exposed animals may suggest a possible role of the cholinergic system in the detrimental effects of Wi-Fi radiation on multisensory integration [100].

Megha et al. [101] exposed rats to microwave radiation at frequencies of 0, 900, 1800 and 2450 MHz (SARs: 0, 0.59, 0.58 and 0.66 mW/kg, respectively) using a transverse electromagnetic cell for 2 months for 2 h/d, 5 d/week. Subsequently, significantly more DNA damage was observed in the exposed groups than in the sham group, and their study also indicated that oxidative stress and inflammation caused DNA damage in response to low-intensity microwave exposure. To investigate whether exposure of rat brains to GMS microwaves induced DNA breaks and changes in gene expression, Belyaev et al. [102] exposed rats to 9.15 MHz microwaves (SAR: 0.4 mW/g) for 2 h. They found that in the cerebellum of all the exposed animals, 11 genes were up-regulated from 1.34- to 2.74-fold, and one gene was down-regulated 0.48-fold. The induced genes encoded proteins with a variety of functions, including neurotransmitter regulation, BBB maintenance, and melatonin production. The study also showed that exposure to GSM microwaves at 915 MHz did not induce detectable DNA double-strand breaks but affected the expression of genes in rat brain cells.

Merola et al. [103] found that microwave radiation caused DNA single-strand and double-strand breaks in vivo in populations submitted to occupational exposure, and the incidence of micronuclei in lymphocytes was significantly increased. However, other reports have indicated that unlike ionizing radiation, the microwave radiation produced by mobile phones does not possess sufficient energy to directly damage DNA. Most bioassay and genotoxicity or mutation studies that have been performed in vitro have reported that exposure to microwave radiation at non-thermal levels does not have mutagenic/genotoxic/carcinogenic effects. Qutob et al. [104] found that exposure to a 1.9 GHz pulse-modulated RF field for 4 h at 0.1, 1.0, and 10 W/kg did not affect gene expression in U87MG glioblastoma cells.

Starting with a single nucleotide polymorphism (SNP) site, Wang et al. [105] identified stable C-T mutation sites at 217 points by screening for SNPs in the GRIN2B promoter region in rats. After exposure to microwave irradiation (an average power density of 30 mW/m^2^ for 5 min/d on five days a week for two consecutive months), the expression of NR2B was decreased in rats, the level of Glu was increased in the hippocampus and cerebrospinal fluid, spatial memory ability was decreased among rats with the TT genotype, and there was no change in the CC type and TC type animals. In the cell experiments, the T allele was significantly more vulnerable to microwaves than the C allele with regard to its transcription factor binding ability and the transcriptional activity and mRNA and protein expression of NR2B. These results explain the genetic mechanisms by which microwave radiation induces damage to learning and memory.

### Energy metabolism

Glucose is the main energy source and is closely related to brain neurotransmitters and cholesterol synthesis [106]. In addition, glucose is also related to cognitive functions, and reductions in the metabolism and uptake of glucose have been observed in local regions of the brain in Alzheimer’s patients [107, 108]. Damage to learning abilities and reduction of glucose utilization in the limbic system of adult rats are closely linked [109]. In the rat hippocampus, glucose uptake plays an important role in spatial learning and memory processes. The rats in the study showed increases in spatial memory and glucose transporters, and this phenomenon indicated a corresponding increase in glucose uptake. In contrast, a central injection of a glucose carrier inhibitor induced injury to memories [110]. Kwon et al. [111] found that the rate of glucose metabolism in the brains of rats was lower after short-term exposure to microwave radiation, and the blood glucose management reduced the damage that was caused by microwave radiation due to the decreased glucose uptake (2.45 MHz, 1 mW/cm^2^, continuous radiation for 30 d at 3 h/d), as were learning and memory capacity. The mechanism by which microwave radiation decreases glucose, resulting in impaired learning and memory in rats, may be related to an increase in the synthesis and release of acetylcholine in the hippocampus. Numerous studies have shown that increased acetylcholine is related to promotion of the effect of glucose on memory [112–114]. Concurrently, in the hippocampus, acetylcholine can promote learning and memory [115, 116]. An increasing level of acetylcholine can increase the concentration of free calcium ions in synapses, but its concentration was decreased by 60% after exposure to microwave irradiation [117]. It has been proposed that by enhancing the functions of acetylcholine, glucose can increase the concentration of free calcium ions in the synapse to reverse microwave-induced damage to learning and memory.

It has been shown that neurons are sensitive to reductions in the availability of adenosine triphosphate (ATP), the main source of energy in mitochondria, which have been reported to be vulnerable to microwave radiation [118]. Microwaves can influence mitochondria by damaging their structure [45], reducing ATP levels and affecting the activity of relevant enzymes such as succinate dehydrogenase (SDH) and cytochrome coxidase (COX) [119–121]. The potential mechanisms underlying these damaging effects range from gene expression alterations in the respiratory chain, membrane damage, Ca^2+^ overloading, and DNA impairment [122–128].

## Summary

It is noteworthy that most of the above mentioned studies were based on the theory that the effects caused by microwaves are non-thermal. However, a recent report [3] published by the Scientific Committee on Emerging and Newly Identified Health Risks (SCENIHR) noted that discussions about the thermal and non-thermal effects are misleading. Because the frequency of microwaves is sufficiently high that the energy is absorbed, the subsequent heating of tissue becomes its major mechanism, and most biochemical and physiological responses are temperature-dependent. Thus, the committee suggested that in the future, studies should explore the border between thermal and non-thermal effects and that specific effects, such as triggering the onset of thermoregulatory reactions, should be defined.

## Conclusion

With the popularity of microwave technology, microwave effects on the human body have become a common topic of concern, and the central nervous system is recognized as a target organ system that is sensitive to microwave radiation. However, to date, with the exception of high-power microwave radiation, which has widely established hazardous effects, the biological effects of microwaves remain controversial. In epidemiology, there is no conclusive evidence showing that microwaves have carcinogenic effects. Concurrently, the discovery that microwaves have positive biological effects has presented new challenges for research and applications in this field. The results of EEG and analyses of the structure of the brain after radiation have also confirmed the influence of microwaves. Studies have extensively explored the underlying mechanisms by which microwaves influence learning and memory functions, especially synaptic structures and functions, oxidative stress and apoptosis, protein synthesis, genes and individual susceptibility and energy metabolism. Previous studies have produced a large amount of information, and some progress has been made in theory, but the mechanisms have not yet been fully determined, and many points are still disputed. The largest problem in these studies is that they used different parameters, such as the frequency, modulation, power density and irradiation time, to apply microwave radiation, in addition to using a variety of research methods. Therefore, their reproducibility and comparability are poor. To determine the precise dose-effect relationship between microwave radiation and its biological effects, further detailed studies must be performed.

## Abbreviations

AMPA: α-amino-3-hydroxy-5-methyl-4-soxazole propionic acid
Asp: Asparaginic acid
ATP: Adenosine triphosphate
BBB: Blood-brain barrier
CaM kinases: Ca^2+^/calmodulin-dependent protein kinases
CMOR: Cross-modal visual-tactile object recognition
COX: Cytochrome coxidase
CTFs: Carboxyl-terminal fragments
DECT: Digital enhanced cordless telecommunications/telephone
DG: Dentate gyrus
EEG: Electroencephalograph
EMF: Electric magnetic field
FR: Frequency radiation
GABA: Gamma-aminobutyric acid
GFAP: Fibrillary acidic protein
Glu: Glutamic acid
Gly: Glycine
GMF: Glial maturation factor
GSM: Global system for mobile communication
IARC: International agency for research on cancer
LTP: Long term potentiation
MAPK: Mitogen-activated protein kinase
MEPSCs: Miniature excitatory postsynaptic currents
MPP: Medial perforant path
MRI: Magnetic resonance imaging
NMDA: N-methyl-D-aspartate
NMDAR: N-methyl-D-aspartic acid receptors
PARP: Poly-ADP-ribose polymerase
PSD-95: Postsynaptic density 95
RNS: Reactive nitrogen species
ROS: Reactive oxygen species
SAR: Specific absorption rate
SCENIHR: Scientific committee on emerging and newly identified health risks
SDH: Succinate dehydrogenase
SNP: Single nucleotide polymorphism
SOR: Spontaneous object recognition
